# A rare case of atypical teratoid rhabdoid tumor at the sellar region in an adult: Case report and review of literature

**DOI:** 10.1016/j.bas.2024.104138

**Published:** 2024-11-14

**Authors:** Georgios Georgountzos, Ioannis Gkalonakis, Georgios Kyriakopoulos, Cleanthi Doukaki, Dimitra Argyro Vassiliadi, Konstantinos Barkas

**Affiliations:** a Department of Neurosurgery, General Hospital of Nikaia ‘Agios Panteleimon’, 18454, Athens, Greece; b Department of Histopathology, General Hospital of Athens ‘Evaggelismos’, 10676, Athens, Greece; c Department of Radiation Oncology, General Hospital of Athens ‘Alexandra’, 11528, Athens, Greece; d Department of Endocrinology, General Hospital of Athens ‘Evaggelismos’, 10676, Athens, Greece

**Keywords:** ATRT, Adult, Sellar, Lung metastasis

## Abstract

**Introduction:**

Atypical teratoid/rhabdoid tumor (AT/RT) of Central Nervous System (CNS) is a rare malignancy, usually confronted in childhood. Although few cases were reported in adults, it seems that there is a preference in supratentorial areas and specifically at the sellar region in middle-aged females with this subgroup presenting distinct features.

**Research question:**

We share a case report of a rare intracranial ATRT in the sellar area with lung metastasis in an adult female.

**Material/methods:**

Patient's medical records (laboratory tests, radiology examinations, histopathology report) were reviewed after retrieving an informed consent. A literature search within Pubmed and further “snowballing” was performed with the use of keywords “sellar”, “adult”, “ATRT” to address the current literature.

**Results:**

We present the case of a 51-year-old woman with headaches and left ptosis, diagnosed with a sellar mass, infiltrating the cavernous sinus. She underwent endoscopic transsphenoidal debulking of the lesion. The pathology report showed an aggressive AT/RT and the patient received radio- and chemo-therapy. On follow up imaging studies, lung metastases were shown and the patient died 7 months after the initial diagnosis.

**Discussion & conclusion:**

Only a few cases of sellar/suprasellar ATRT with lung metastases have been described so far. ATRT should be in the differential diagnosis of fibrous sellar masses in adult women.

## Highlights

• Sellar ATRT is a rare malignancy in adults, mostly affecting middle-aged women.
• Molecular testing and immunohistochemistry for loss of nuclear INI1 remain the mainstay for the diagnosis.
• Extracranial dissemination may occur and worsen the overall poor prognosis of the disease.
• Multimodal approach with maximal surgical resection, radiotherapy and chemotherapy seem to improve overall survival.

## Abbreviations

AT/RT: Atypical Teratoid/Rhabdoid Tumor
CN: Cranial Nerve
CNS: Central Nervous System
CSF: Cerebrospinal Fluid
EMA: Epithelial Membrane Protein
ETSS: Endoscopic Transnasal Transsphenoidal Surgery
F: Female
FSH: Follicle Stimulating Hormone
GFAP: Gliofibrillary acid protein
Gy: Gray
GTR: Gross Total Resection
H/A: Headache
IGF-1: Insulin-like Growth Factor 1
LH: Luteinizing hormone
M: Male
MRI: Magnetic Resonance Imaging
N/A: Not Available
NR: Not Reported
PR: Partial Resection
RT: Radiotherapy
SALL-4: Sal-like Protein 4
SD: Standard Deviation
SMA: Smooth Muscle Actin
STR: Subtotal Resection
TSH: Thyroid Stimulating Hormone
VIM: Vimentin
WHO: World Health Organization

## 1 Introduction

Atypical teratoid/rhabdoid tumors (AT/RT) are an uncommon and highly aggressive form of malignancy characterized by histological traits resembling both rhabdoid and teratoid cells, as indicated by its name. It was first described in 1981 by Biggs et al. and was included in the World Health Organization (WHO) Classification of 2000 (Biggs et al., 1987; Kleihues et al., 2002).

Sellar tumors account for 14–18% of intracranial tumors (Al-Dahmani et al., 2015). It seems that sellar ATRT represents a distinct entity with altered histopathologic and genetic characteristics (Chan et al., 2018; Johann et al., 2018; Nakata et al., 2017). Typically, it occurs in childhood and more frequent in males 0–3 years old (Rorke et al., 1996). AT/RT of CNS in adults is extremely rare with less than 100 cases described (Major et al., 2022; Nakata et al., 2017). Typically, it manifests within the cerebral hemispheres and the sellar region, in contrast to pediatric ATRT which primarily occurs in the posterior fossa. (Wu et al., 2016).

The fundamental basis for diagnosis rests upon the identification of the loss of nuclear expression of SMARCB1/INI1 which is recognized through immunohistochemistry and/or molecular studies with partial deletion of chromosome 22 along with histological findings such as rhabdomyoid cells and staghorn vasculature (Louis et al., 2021). Similar changes may also occur in other types of primary CNS tumors as they progress over time. So far, 8 cases of AT/RT (or INI1-negative rhabdoid tumors) arising in the setting of other primary CNS tumors have been reported in the literature (2 in gangliogliomas, 3 in pleiomorphic xanthoastrocytoma, and 3 in high-grade gliomas) (Nobusawa et al., 2016).

The primary treatment approach involves a combination of surgery, radiotherapy, and chemotherapy. However, there are currently no standardized therapeutic protocols for adults and in clinical practice approaches used in pediatric cases are often employed. While adjuvant treatment types varied greatly between individuals, those who underwent adjuvant chemotherapy and radiation therapy had significantly longer survival times compared to those who did not (Broggi et al., 2022; Major et al., 2022). There are no definitive guidelines for optimal treatment, and the prognosis of this tumor is poor with a median survival of approximately 1 year.

Herein, we present a new case of sellar ATRT with metastases who underwent combination therapy and a literature review of the cases reported so far.

## 2 Methods

After patient's written consent, medical records including imaging studies, laboratory tests, histopathology report and follow-up notes were reviewed.

**Fig. 1:**
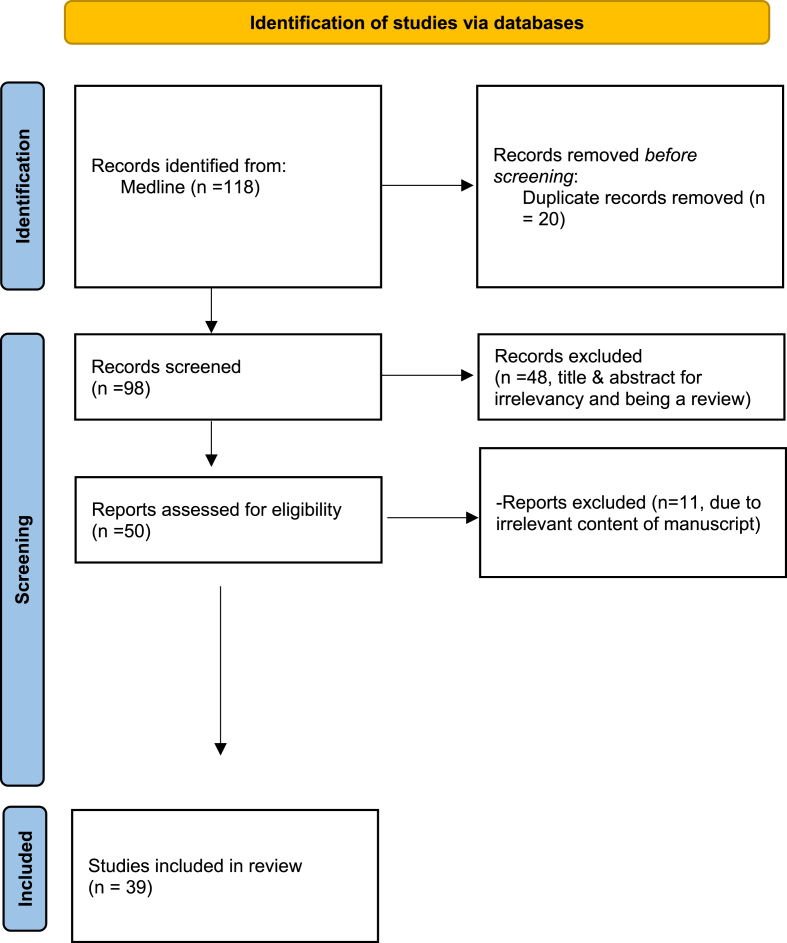
PRISMA flow diagram of our literature search.

In our effort to compile the published cases of adult sellar ATRTs, we conducted a comprehensive search in PubMed database, along with “snowballing” from reference lists of related articles, using keywords terms as “sellar”, “parasellar”, “adult”, “atypical teratoid rabdoid tumor”. The flowchart of our literature search is presented in Fig. 1. Statistical analysis with descriptive statistics and Kaplan-Meier analysis for survival between groups were performed with the use of SPSS software (IBM Corp. Released, 2019. IBM SPSS Statistics for Windows, Version 26.0. Armonk, NY: IBM Corp).

## 3 Results-case presentation

A 51-year-old right-handed Caucasian woman with no past medical history, was admitted to our neurosurgical department in February 2020 due to a sella turcica mass. Over the past five months, she had been experiencing progressively severe headaches, and in the last two weeks, she had also observed left ptosis. She denied any other symptoms such as galactorrhea, menstruation disturbances or convulsions.

On neurological examination, she suffered from left III (oculomotor) and IV (trochlear) nerve palsies. Her headaches were not controlled despite the use of opioids. The patient underwent a comprehensive hormone profile assessment, revealing ACTH deficiency indicated by a low serum cortisol level (6 μg/dl). Subsequently, she was initiated on replacement therapy with hydrocortisone. No indications of additional pituitary hormone deficits were detected, as hormone levels encompassing prolactin, TSH, FT4, IGF-1, FSH, LH, and estradiol were all within the expected ranges. Moreover, there were no signs of diabetes insipidus.

**Fig. 2:**
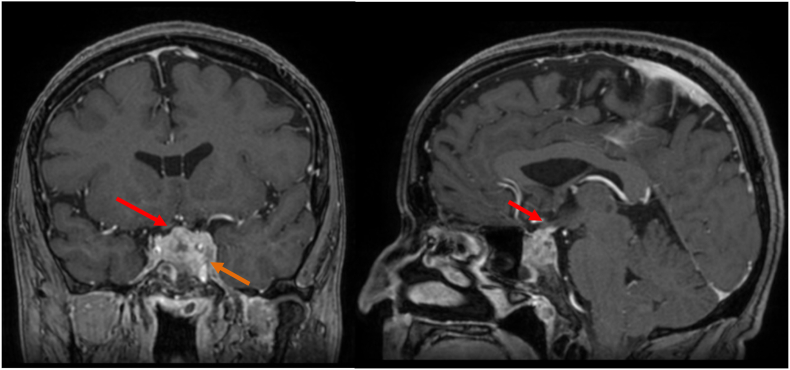
Gadolinium-enhanced T1 sequence in coronal (left) and sagittal (right) view showing the heterogenous enhancement of the sellar mass with pressure of the optic chiasm (red arrow) and infiltration of clivus and the left cavernous sinus wall with carotid encasement (orange arrow). (For interpretation of the references to colour in this figure legend, the reader is referred to the Web version of this article.)

On preoperative imaging with MRI, a large mass of the sellar region with compression of the optic chiasm and infiltration of the left cavernous sinus was identified as seen on Fig. 2.

**Fig. 3:**
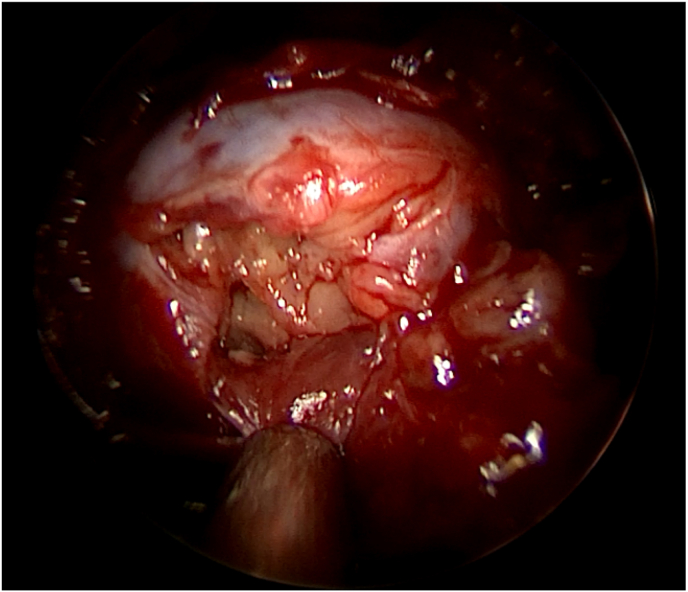
Intraoperative view of the sellar mass as seen from ETSS, when the dura is opened. The mass was fibrous and difficult to dissect. A decision to debulk and take biopsies was made, respecting the high risk of vascular or neural damage to adjacent anatomical structures.

The working diagnosis was that of a pituitary apoplexy in the background of a non-functioning macroadenoma and due to the severity of the headache an operation was offered to the patient. The sellar area tumor was approached via an endoscopic transnasal transsphenoidal surgery (ETSS). Intraoperatively a robust, hemorrhagic and significantly fibrous mass was identified, not compliant with the usual texture of pituitary adenoma (Fig. 3). We were not able to significantly debulk the tumour due to its texture so only a small amount of tumor was removed and was sent to the pathology department for histopathology exam. The patient had an uneventful recovery with resolution of her headaches. The cranial nerve palsies were not improved as expected. She was discharged on hydrocortisone replacement.

**Fig. 4:**
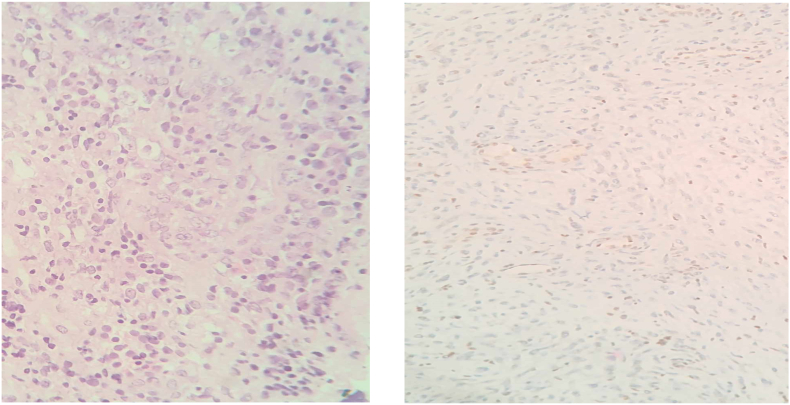
Medium sized neoplastic cells with epithelioid/rhabdoid morphology on hematoxylin/eosin staining (left, x400). Immunostaining (right, x400) showing typical loss of nuclear expression of SMARCB1/INI-1 in the neoplastic cells. Retain of the protein expression in the endothelial cells (positive internal control).

The histopathology report referred to a poorly differentiated (grade IV) neoplasm with medium to large-sized cells with vesicular nuclei and prominent nucleoli, and pale, clear cytoplasm (Fig. 4). The specimen was tested negative immunohistochemically for mesenchymal, neuronal, neuroendocrine, glial, melanoma, haemopoietic, keratin and other markers. The complete loss of nuclear expression of SMARCB1/INI1 (BAF47 clone) along with the positive germ cell marker SALL-4+ set the diagnosis of the atypical teratoid/rhabdoid tumor. The ki-67 index was found 50–60% which pointed out the aggressiveness of the tumor.

The patient was referred to an oncology department where she underwent whole neuraxis, chest/abdominal CT imaging for disease staging (negative for secondary foci) and received combined chemotherapy with cisplatin and etoposide, and whole brain radiotherapy with VMAT protocol of 60 Gy in 30 fractions. Four months postoperatively, she was found with mediastinal lymph node infiltration and pulmonary metastases. She passed away 3 months later due to respiratory failure.

## 4 Discussion

ATRT is a highly aggressive malignant tumor, occurring mostly in infancy. Adult ATRT remains a rare entity with distinct characteristics and its diagnosis is based on the inactivation of SMARCB1 (INI1) or SMARCA4 (BRG1). A literature search regarding sellar ATRT cases in Pubmed was initiated, as mentioned in Methods. The results are summarized in Table 1.

**Table 1:** Cases of sellar ATRT.

Author, Year	Age, Sex	Clinical symptoms	Cavernous sinus invasion	Ki-67%	Extent of resection	Adjuvant Treatment	Lung Dissemination	Outcome
Kuge et al., 2000	32, F	H/A, Visual Disturbance	N	NR	STR	Chemo + RT	NR	Alive, 28 mos
Raisanen et al., 2005	31, F	–	N	NR	NR	RT	NR	Dead, 9 mos
Raisanen et al., 2005	20, F	Visual Disturbance	Y	NR	NR	Chemo + RT	NR	Alive, 28 mos
Arita et al., 2008	56, F	H/A, Visual Disturbance, CN palsy	Y	30%	STR	RT	NR	Dead, 23 mos
Las Heras and Pritzker, 2010	46, F	H/A	NR	NR	NR	NR	NR	NR
Schneiderhan et al., 2011	61, F	CN palsy	Y	50%	STR	–	NR	Dead, 3 mos
Schneiderhan et al., 2011	57, F	H/A, Visual Disturbance, CN palsy	Y	80%	GTR	Chemo + RT	NR	Alive, 6 mos
Moretti et al., 2013	60, F	H/A, Visual Disturbance, CN palsy	Y	25%	STR	Chemo + RT	**Y**	Dead, 30 mos
Chou et al., 2013	43, F	H/A, Visual Disturbance	Y	NR	STR	RT	NR	Alive, 2 weeks
Park et al., 2014	42, F	Visual Disturbance	Y	NR	STR	Chemo + RT	NR	Alive, 27 mos
Shitara and Akiyama, 2014	44, F	Visual Disturbance, Nerve palsy	Y	>90%	PR X2	Chemo + RT	**Y**	Dead, 17 mos
Lev et al., 2015	36, F	H/A, Visual Disturbance, CN palsy	Y	NR	STR X3	Chemo + RT	NR	Dead, 30 mos
Regan et al., 2015	45, F	H/A, Visual Disturbance, CN palsy	Y	NR	STR	RT	NR	Dead, 6 mos
Biswas et al., 2015	48, F	Visual Disturbance	NR	NR	STR X2	Chemo + RT	NR	Dead, 2 mos
Larrán-Escandón et al., 2016	43, F	H/A, Visual Disturbance, CN palsy	Y	NR	PR	–	NR	Dead, 1 mo
Nobusawa et al., 2016	69, F	Visual Disturbance, CN palsy	Y	60%	STR	Chemo + RT	NR	Alive, 24 mos
Elsayad et al., 2016	66, **M**	H/A, CN palsy	Y	NR	STR, GTR	RT	NR	Alive, 48 mos
Almalki et al., 2017	36, F	H/A, Visual Disturbance, CN palsy	Y	NR	STR	Chemo + RT	NR	Alive, 36 mos
Dardis et al., 2017	35, **M**	Visual Disturbance	NR	NR	STR	Chemo + RT	NR	Alive, 30 mos
Nakata et al., 2017	26, F	NR	NR	30%	NR	Chemo + RT	NR	Dead, 33 mos
Nakata et al., 2017	21, F	NR	NR	20%	NR	Chemo + RT	NR	Dead, 35 mos
Pratt et al., 2017	47, F	H/A	Y	>50%	STR	RT	NR	NR
Barresi et al., 2018	59, F	H/A, Visual Disturbance, CN palsy	Y	70%	STR	RT	NR	Dead, 2 mos
Nishikawa et al., 2018	42, F	H/A, Visual Disturbance, Vertigo, CN palsy	Y	30%	STR X2	Chemo + RT	NR	Dead, 11 mos
Johann et al., 2018	66, **M**	NR	NR	NR	NR	NR	NR	Alive, 54 mos
Johann et al., 2018	20, F	NR	NR	NR	NR	Chemo + RT	NR	Dead, 120 mos
Johann et al., 2018	48, F	NR	NR	NR	NR	NR	NR	Alive, 4 mos
Johann et al., 2018	46, F	NR	NR	NR	STR	–	NR	Dead, Postop
Paolini et al., 2018	65, F	NR	NR	NR	STR	Chemo + RT	NR	Dead, 23 mos
Paolini et al., 2018	47, F	NR	NR	NR	STR	Chemo + RT	NR	Alive, 62 mos
Paolini et al., 2018	31, F	NR	NR	NR	STR	–	NR	Dead, 2mos
Paolini et al., 2018	36, F	NR	NR	NR	STR	Chemo + RT	NR	Alive, 22 mos
Barsky, 2018	54, F	Fatigue, weight loss, CN palsy	Y	50%	STR	NR	NR	NR
Su and Su, 2018	37, F	Visual disturbance	NR	60%	STR	–	NR	Dead, 2 mos
Asmaro et al., 2019	62, F	H/A, Visual Disturbance, CN palsy	Y	NR	STR	–	NR	Dead, <2 mos
Ahmad et al., 2019	33, F	H/A, CN palsy	Y	30%	STR	Chemo + RT	NR	Alive, 36 mos
Voisin et al., 2019	51, F	Visual Disturbance	NR	7%	STR	Chemo + RT	NR	Alive, 9 mos
Siddiqui et al., 2019	55, F	H/A, Visual Disturbance, Altered mental status	Y	NR	STR X2	–	NR	Dead, 1.5 mos
Lawler and Robertson, 2019	27, F	Galactorrhea, Visual Disturbance	NR	30%	STR	NR	NR	NR
Liu et al., 2020	43, F	H/A, Visual Disturbance, CN palsy	NR	20%	STR	Chemo + RT	NR	Dead, 4 mos
Liu et al., 2020	52, F	H/A, limb numbness	NR	50%	STR	–	NR	Dead, 2 mos
Liu et al., 2020	50, F	H/A, visual disturbance limb numbness, galactorrhea	NR	40%	GTR	–	NR	Dead, 1 mo
Liu et al., 2020	29, F	H/A	NR	30%	STR	RT	NR	Dead, 8 mos
Liu et al., 2020	80, F	H/A, Visual Disturbance, CN palsy	NR	50%	STR	–	NR	Dead, 1 mo
Bokhari et al., 2020	40, F	H/A, Visual Disturbance	Y	NR	STR	Chemo + RT	NR	Dead, 1 mo
Oraibi et al., 2020	70, F	H/A	NR	60%	STR	Chemo + RT	NR	Dead, 6 mos
FUKUDA et al., 2021	45, F	Visual Disturbance, CN palsy	Y	70%	STR	Chemo + RT	**Y**	Dead, 5 mos
Peng et al., 2021	43, F	H/A, Visual Disturbance, CN palsy	Y	NR	PR	RT	NR	Dead, 4 mos
Peng et al., 2021	52, F	H/A, limb numbness	NR	NR	PR	–	NR	Dead, 2mos
Peng et al., 2021	50, F	H/A, Visual Disturbance, galactorrhea, seizure	NR	NR	GTR	–	NR	Dead, 1 mo
Peng et al., 2021	29, F	H/A	NR	NR	PR	Chemo + RT	NR	Dead, 8 mos
Peng et al., 2021	80, F	H/A, Visual Disturbance, CN palsy, lethargy	Y	NR	PR	–	NR	Dead, 1 mo
Mhatre et al., 2021	33, F	N/A	N/A	N/A	N/A	N/A	N/A	N/A
Major et al., 2022	70, F	H/A, Visual Disturbance, CN palsy	Y	60%	STR	Chemo + RT	NR	Dead, 5.5 mos
Baiano et al., 2022	32, F	H/A, Visual disturbance, polyuria, polydipsia, CN palsy	Y	90%	STR	Chemo	NR	Dead, 2mos
Baiano et al., 2022	40, F	H/A, Visual Disturbance, CN palsy	Y	>40%	GTR	Chemo	NR	Dead, 2 mos
Baiano et al., 2022	41, F	Panhypopituitarism, polyuria, polydipsia	Y	40%	STR	–	NR	Dead, 1 mo
Baiano et al., 2022	50, F	H/A, polyuria, polydipsia,	Y	>40%	STR	Chemo + RT	NR	Alive, 18 mos
Aldhafeeri et al., 2023	32, **M**	H/A, Altered mental status, seizure	Y	10%	STR	–	NR	Dead, 3mos
Current case	51, F	H/A, Visual Disturbance, CN palsy	Y	60%	PR	Chemo + RT	**Y**	Dead, 7 mos

Abbreviations: Chemo: Chemotherapy, CN: cranial nerve, F: Female, GTR: gross total resection, H/A: Headache, M: Male, mos: months, RT: Radiotherapy, N/A: not available, NR: not reported, PR: partial resection, STR: subtotal resection.

### 4.1 Patient demographics and clinical characteristics

Sellar ATRT has never been reported in children and shows a clear female predominance with only 4 male patients having been reported (Aldhafeeri et al., 2023; Dardis et al., 2017; Elsayad et al., 2016; Johann et al., 2018). Our search in PubMed found, 39 related articles, reporting sixty patients (including our patient) with mean age of 45.92 years (SD: 14.2 years, Range: 20–80 years) of which 93.3% (n = 56) were female. Chan *et al* and Broggi *et al* also reported a similar age group with mean age 36.7–38 (Broggi et al., 2022; Chan et al., 2018). Our case adds up to the female cases of sellar ATRT, confirming the gender pattern stated by previous investigators, while other adult and pediatric ATRT seem to have male predominance (Barresi et al., 2018; Chan et al., 2018). The background of female predominance is yet to be clarified. Barresi et al. (2018) and Peng et al. (2021) speculated that there might be a relation with estrogens and their receptors. On the other hand, the fact that ATRT presents mostly in middle age and not older females, like breast cancer, takes away the theory of hormonal relation (Asmaro et al., 2019). Also, patterns of female predisposition of specific tumors have been reported in non-sex related organs such as pancreas and kidney (Nakata et al., 2017).

Until 2014, adult ATRT was reported mostly in cerebral hemispheres followed by sellar region (Ostrom et al., 2014). Lately, it seems that sellar region (41.9–46.9%) has overcome cerebral hemispheres (21.9–35.14%) with pineal region, cerebellopontine angle, cerebellar hemisphere, spine following (Broggi et al., 2022; Chan et al., 2018; Peng et al., 2021). Our case adds up to the growing incidence of sellar region ATRTs.

In comparison to pituitary macroadenomas, ATRT of the sella typically present with persistent headaches and visual disturbances. Pituitary macroadenomas, too, may exhibit visual disturbances and headaches if the tumor compresses optic nerves or adjacent structures. However, AT/RT cases may feature additional symptoms stemming from the tumor's aggressive nature, including neurological deficits related to infiltration of surrounding tissues. This is an atypical manifestation in pituitary macroadenomas unless they undergo apoplexy and this is the primary reason behind our initial diagnosis in this particular case. Pituitary macroadenomas often associate with hormonal disturbances, either through hypersecretion, in cases of functioning tumors, or by causing hypopituitarism, leading to a range of diverse symptoms. Although hormonal imbalances can arise in AT/RT cases, they generally do not serve as the primary hallmark of this malignancy. Instead, the emphasis often lies on the aggressive behavior of the tumor and its impact on adjacent anatomical elements. (Baiano et al., 2022; Major et al., 2022). Similarities are also found in radiologic findings of these tumors and macroadenomas with heterogenous enhancement in gadolinium enhanced T1 sequence being the most common and non-specific (Bokhari et al., 2020). There are also 3 reported cases presenting with signs and symptoms of intracranial hypertension due to intraventricular and/or subarachnoid haemorrhage (Aldhafeeri et al., 2023; Asmaro et al., 2019; Siddiqui et al., 2019).

### 4.2 Histological and molecular characteristics

Cellular features of these tumors vary from small to large cellular infiltrate, usually with clear cytoplasm and prominent nucleoli with characteristic rhabdoid cells being also reported in some cases. A vasculature pattern described as staghorn has also being proposed by Nakata et al. as being a feature only of sellar ATRTs (Nakata et al., 2017). Immunohistochemistry is of great importance in the differential diagnosis of these tumors with loss of nuclear INI1 staining being the cornerstone (Paolini et al., 2018; Voisin et al., 2019) and vimentin (VIM), smooth muscle actin (SMA), epithelial membrane protein (EMA), chromogranin and gliofibrillary acid protein (GFAP) acting ancillary (Baiano et al., 2022; Barresi et al., 2018). In our case, complete loss of INI1, with EMA negative, SMA negative, GFAP negative, chromogranin negative and sal-like protein 4 (SALL-4) positive guided our diagnosis towards ATRT rather than dedifferentiated/poorly differentiated chordoma. Different genetic alterations leading to loss of INI1 expression, seem also to distinguish pediatric and adult ATRT from sellar ATRT as previously reported (Aldhafeeri et al., 2023; Nakata et al., 2017; Nishikawa et al., 2018). Although, most cases of adult ATRT so far are due to sporadic mutations of the SMARCB1/INI1/hSNF5 gene, Voisin et al. in his study reported a case of a familial mutation resulting in adult sellar ATRT (Voisin et al., 2019), underlining the importance of genetic testing in diagnosis of these tumors. According to the literature there are three methylation profiles of pediatric ATRT. A study of Johann et al. (2018), also endorsed by other researchers (Baiano et al., 2022; FUKUDA et al., 2021) proposed that sellar ATRT in adults resembles the ATRT-MYC pediatric profile which gives the opportunity of therapy individualization and supports the notion of sellar ATRT being a unique entity. Ki-67% is a well-established marker of cell proliferation as well as of tumor growth, used in the prognosis of the disease. As in other tumors, high levels of ki-67% (>35%) is correlated with worse outcome in sellar ATRT as shown by Liu et al. (2020) and a recent meta-analysis of Broggi et al. (2022). In our case, Ki-67% was 60% with median Ki-67% being 50% in our literature review (Range 7–90%), although it seems that in many cases this marker was not determined (32 patients missing data). Knowing this information, it is of outmost importance of determining any marker that may serve as a predictor of outcome and may guide the therapeutic modality.

### 4.3 Prognosis

As in pediatric population, adult sellar ATRT is characterized by poor prognosis. The mean overall survival was 15.44 months and ranged from 0 to 120 months (SD: 20.95 months), while 5 studies reported no survival data. On follow-up, 29% (16/55) of the patients were alive whilst the majority (n = 39/55) has deceased. Sellar ATRT seems to have better overall survival than pediatric and other location adult ATRT as reported by well conducted reviews (Chan et al., 2018; Nakata et al., 2017). Major et al. (2022) reported a median overall survival of 19.5 months in patients with sellar ATRT vs 15 months in patients with CNS ATRT according to Broggi et al. (2022), indicating a more favorable outcome in sellar ATRT. Although according to our review they seem similar and further assessment with multi-center studies is needed.

The most common approach for these tumors is the transsphenoidal approach. Sellar ATRTs, as aggressive tumors, very often invade adjacent structures as the cavernous sinus (n = 32/60), clivus and internal carotid artery and have unclear cleavage planes making their total excision a difficult task. As we observed from the literature, in many patients complete excision (n = 5/51) was not feasible while in 90.6 % (n = 48/51) of cases including ours, partial resection for decompression and biopsy was performed with the difference between partial and subtotal resection being obscure (Peng et al., 2021). Also, there were 7 cases with no specific data regarding the extent of resection (Johann et al., 2018; Raisanen et al., 2005). While in pediatric population there is clear superiority of GTR vs STR and PR in overall survival, in adults there is still no statistical significance supporting that notion, although two studies reported difference between GTR and STR (Chan et al., 2018; Major et al., 2022), delineating the need for multicenter studies.

**Fig. 5:**
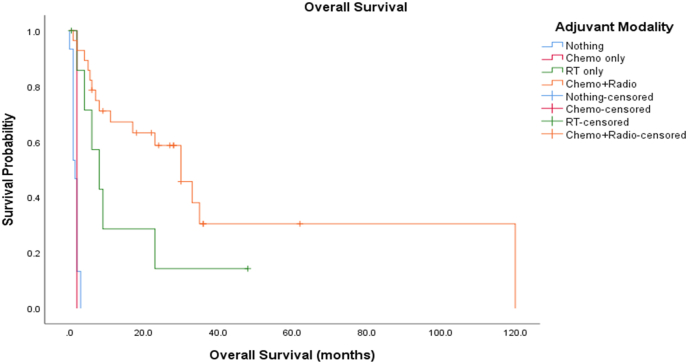
Kaplan-Meier survival curves for different adjuvant treatment options. Combination of chemotherapy and radiotherapy showed statistically significant difference in overall survival vs other options *(log rank test, p-value< 0.05)*.

Adjuvant chemotherapy consists of multi-regimen chemotherapy and focal and/or craniospinal radiotherapy, based mostly on children's protocols. Chemotherapy regimens vary a lot with combinations of vincristine, doxorubicin, cyclophosphamide or ifosfamide, carboplatin, and etoposide being commonly chosen. According to our review, management only with chemotherapy regimens was chosen only in 2 cases. In 15 patients no further adjuvant treatment was administered. The majority of patients (51.9%) with sellar ATRT received protocols combining chemotherapy and radiotherapy. As shown in Fig. 5, combination of chemotherapy and radiotherapy has been shown to be associated with better survival and its early initiation is very important in prognosis, as mentioned already in the literature (Chan et al., 2018; Major et al., 2022). Recently a protocol conducted by Slavc et al. including three 9-week courses of a dose-dense regimen including doxorubicin, cyclophosphamide, vincristine, ifosfamide, cisplatin, etoposide, and high-dose methotrexate with intrathecal therapy followed by high-dose chemotherapy and sub-sequent focal radiotherapy showed promising results with 5-year survival rate of 100% in nine cases of ATRT (Slavc et al., 2014). Further clinical trials are needed to clarify the optimal combination of chemotherapy regimen for adults. Our understanding of approaching this entity is to address this tumor initially with maximal safe resection and by the time the histopathology results are available, aggressive chemotherapy and radiotherapy should be initiated.

### 4.4 Lung metastasis

In ATRT patients, dissemination is observed either on diagnosis or at some point over the course of the disease. As previously reported, although many patients do not receive whole neuroaxis scan, local dissemination and CSF dissemination is well described (Chan et al., 2018; Peng et al., 2021). However, extraneural dissemination is extremely rare with only 3 cases being reported with distant dissemination in lungs (FUKUDA et al., 2021; Moretti et al., 2013; Shitara and Akiyama, 2014). To the best of our knowledge our case is the fourth case with lung metastasis. We speculate that this happens due to cavernous sinus invasion and maybe there are more cases with lung dissemination in the current literature that may be misdiagnosed, as also reported by Fukuda et al. (FUKUDA et al., 2021). These patients must rapidly get involved in chemotherapy and radiation protocols for better prognosis.

## 5 Conclusion

Adult sellar ATRT seems to form a distinct subcategory of its own amongst CNS ATRTs with clinical and pathological characteristics. To the best of our knowledge, this is the fourth case of sellar ATRT with lung dissemination in the literature. It remains an extremely rare entity, that should be in the differential diagnosis of invasive pituitary mass and whole neuraxis and extraneural staging imaging must be in the work-up tests when initial diagnosis is made. Due to the rarity of the cases, multicenter, well-established studies are needed in order to define the optimal combination treatment protocol.

## Authorship confirmation statement

All authors contributed to the study conception. The data collection and analysis were performed by G. Georgountzos, C. Doukakis, Vassiliadi D. A. and Gkalonakis I. Mr Barkas K. and Ms Vassiliadi D. A. made the revision the manuscript. All authors read and approved the final manuscript. An informed consent of the patient was obtained.

## Authors’ disclosure statement

The authors declare that they have no competing interests.

## Declaration of competing interest

The authors declare that they have no known competing financial interests or personal relationships that could have appeared to influence the work reported in this paper.
